# Scientific Approaches on Extraction, Purification and Stability for the Commercialization of Fucoxanthin Recovered from Brown Algae

**DOI:** 10.3390/foods9081113

**Published:** 2020-08-13

**Authors:** Catarina Lourenço-Lopes, Paula Garcia-Oliveira, Maria Carpena, Maria Fraga-Corral, Cecilia Jimenez-Lopez, Antia G. Pereira, Miguel A. Prieto, Jesus Simal-Gandara

**Affiliations:** 1 Nutrition and Bromatology Group, Analytical and Food Chemistry Department, Faculty of Food Science and Technology, University of Vigo, Ourense Campus, E-32004 Ourense, Spain; c.lopes@uvigo.es (C.L.-L.); paula.garcia.oliveira@uvigo.es (P.G.-O.); maria.carpena.rodriguez@uvigo.es (M.C.); mfraga@uvigo.es (M.F.-C.); cecilia.jimenez.lopez@uvigo.es (C.J.-L.); antia.gonzalez.pereira@uvigo.es (A.G.P.); 2 Centro de Investigação de Montanha (CIMO), Instituto Politécnico de Bragança, Campus de Santa Apolonia, 5300-253 Bragança, Portugal

**Keywords:** brown macroalgae, extraction, fucoxanthin, purification, quantification

## Abstract

The scientific community has corroborated the numerous beneficial activities of fucoxanthin, such as its antioxidant, anti-inflammatory, anticancer or neuroprotective effects, among others. These properties have attracted the attention of nutraceutical, cosmetic and pharmacological industries, giving rise to various possible applications. Fucoxanthin may be chemically produced, but the extraction from natural sources is considered more cost-effective, efficient and eco-friendly. Thus, identifying suitable sources of this compound and giving a general overview of efficient extraction, quantification, purification and stabilization studies is of great importance for the future production and commercialization of fucoxanthin. The scientific research showed that most of the studies are performed using conventional techniques, but non-conventional techniques begin to gain popularity in the recovery of this compound. High Performance Liquid Chromatography (HPLC), Nuclear Magnetic Resonance (NMR) and spectroscopy techniques have been employed in the quantification and identification of fucoxanthin. The further purification of extracts has been mainly accomplished using purification columns. Finally, the stability of fucoxanthin has been assessed as a free molecule, in an emulsion, or encapsulated to identify the variables that might affect its further industrial application.

## 1. Introduction

Traditionally, marine resources like micro- and macroalgae have been used as foods and medicines, mainly in oriental countries, such as China, Japan or Indonesia. However, their popularity is growing in western countries. In previous years, numerous studies have reported that these organisms present high nutritional values as foods and could be a possible source of compounds with bioactive potential. Several examples are fatty acids, carotenoids, polysaccharides, phytosterols, and phenolic compounds, which have been demonstrated to exert beneficial health effects, such as anticoagulant, antitumor or antioxidant properties [1]. Thus, the development of new formulations in food, cosmetic and pharmaceutical sectors using an algae compound is gaining relevance. Among macroalgae, brown ones (Class Phaeophyta) have been reported to present a greater amount of bioactive compounds, compared with red (Class Rhodophyta) and green (Class Chlorophyta) macroalgae [2]. Some bioactive compounds of brown macroalgae stand out such as phlorotannins, phylopheophylin and specially fucoxanthin [3]. Fucoxanthin is a secondary metabolite that belongs to the family of carotenoids and is present in the chloroplasts of algae cells. It is considered one of the most abundant and representative pigments of brown algae. This compound has been studied in numerous brown algae, such as in the genera *Undaria*, *Sargassum*, *Laminaria*, *Eisenia*, *Alaria*, *Cystoseira or Hijikia* [4,5], but it has also been found in red, green and unicellular microalgae [6,7,8].

Numerous scientific works have corroborated the beneficial activities of fucoxanthin, including antioxidant, anticancer, antihypertensive, anti-inflammatory, anti-diabetic, anti-obesity, neuroprotective, anti-angiogenic capacities and also photoprotective effects [4,7,9,10,11,12,13,14,15,16]. Considering these properties, the fucoxanthin molecule has a potential application in several industrial sectors such as food, cosmetic and pharmaceutical sectors. In fact, an expansion of the fucoxanthin market that might reach $120 million by 2022 is expected [17]. Nevertheless, the commercialization and production of this compound still have to face certain challenges, since its chemical synthesis is a complex and inefficient process, and the method of extraction from marine organisms has not been yet standardized [18,19]. To achieve the profitably of a product, it should be easily and quickly obtained, using low-cost technologies [20]. Although fucoxanthin and other carotenoids may be artificially synthesized, their extraction from natural sources presents several advantages, such as easy accessibility to the sources and an economic and environmentally friendly production, avoiding the use of dangerous chemical compounds. Consequently, future and innovative studies about extraction methods would be of great interest to favor the commercialization of fucoxanthin.

Several studies have cultivated brown algae to extract fucoxanthin, and many aspects have been demonstrated to influence its content, including environmental factors (water temperature, composition, light, etc.), stage of the life cycle or seasonal variations. *Undaria pinnatifida* is recognized as a key species to produce fucoxanthin and has been widely cultured in oriental countries (mostly in Japan, Korea and China), but its culture is growing in western ones. This species has been cultivated in deep seawater, due to several advantages, like the abundance of nutrients and low presence of pathogens. In this situation, the fucoxanthin content ranged between 0.32–2.67 mg/g, being lower in the later stages than in the younger stages of the life cycle [21]. In another study, *U. pinnatifida* was collected from two localizations during the growing season. The variations in the fucoxanthin content between localizations were attributed to environmental factors, such as sunlight, temperature and the amount of nutrients present in the water. In both locations, the content peaked between winter and spring, while the lowest peak was detected in summer. In addition, the blade of the algae (structure used for food applications) showed a higher fucoxanthin content, compared with sporophyll (reproductive structure, not consumed) [22]. Similar spatial and seasonal variations have been observed in other brown algae species such as *Sargassum horneri*, *Cystoseira hakodatensis* and *Nizamuddinia zanardinii* [23,24,25].

**Figure 1:**
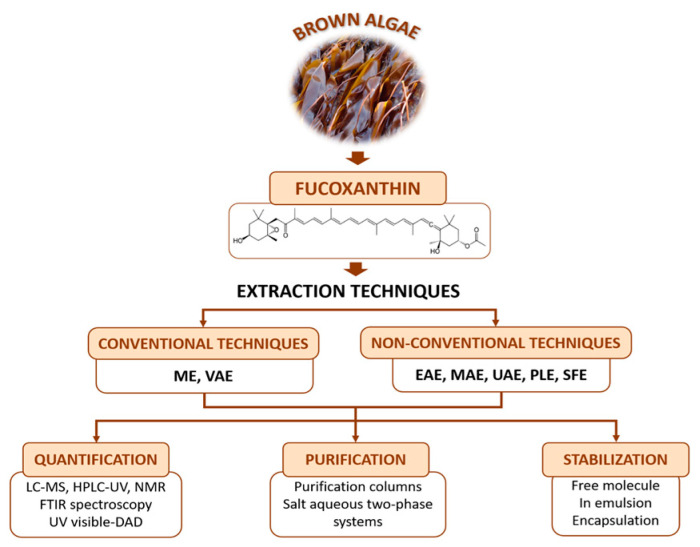
General overview of the sources of fucoxanthin, extraction and quantification/identification techniques, purification systems and stabilization studies for further industrial application of this compound. The definition for the abbreviations used in this figure can be seen in the abbreviation list provided. Extraction methods and conditions: Maceration extraction (ME); Vortex-assisted solid-liquid micro-extraction (VAE); Enzyme assisted extraction (EAE); Microwave-assisted extraction (MAE); Ultrasound-assisted extraction (UAE); Pressurized liquid extraction (PLE); Supercritical fluid extraction (SFE). **Detection method:** High Performance Liquid Chromatography (HPLC); HPLC with diode array detector (HPLC-DAD); HPLC with ultraviolet detector (HPLC-UV); Liquid Chromatography-Mass Spectrometry (LC-MS); Nuclear Magnetic Resonance (^1^H-NMR); Fourier Transform-Infrared Spectroscopy (FTIR).

Considering all the factors that influence the fucoxanthin content, the obtaining process should be optimized to obtain the highest wield possible. The present study aims to provide a full vision of extraction, quantification, and purification methods that will allow the choice of the optimal protocol for recovering the highest ratio of fucoxanthin depending on the selected species and the laboratory facilities available. Also, three stabilization methods of the molecule will be reviewed, due to the importance of preventing fucoxanthin losses induced by degradation reactions. A general overview is presented in Figure 1.

## 2. Extraction Methods

Scientific literature shows several fucoxanthin extraction techniques employed to improve the extraction yields and to reduce costs. In this section, the methods will be presented in terms of yield. Table 1 compiles an extended list of brown algae in which fucoxanthin has been extracted using conventional and non-conventional techniques, respectively. In addition, the detection methods and their concentrations are also mentioned.

### 2.1. Conventional Techniques

#### 2.1.1. Maceration Extraction (ME)

Most of the developed techniques found throughout the literature for obtaining fucoxanthin are based on ME protocols, which have been historically used for common extraction processes. This technique consists of a solid/liquid extraction in which different variables are evaluated, such as biomass-solvent ratio, percentage of solvent, time and temperature of incubation [26]. The biomass: solvent ratio used for optimizing the protocols has been observed to vary a lot with wide ranges going from 1:10 up to 1:500 [27,28]. Temperature and time of extraction are also very variable. The controlled temperatures tested for ME ranged from 4 °C up to 65 °C or in ice baths or at room temperatures, while evaluated times have ranged from 15 min up to 96 h. Besides, when extracting fucoxanthin, the choice of solvent is very important since it determines the efficiency of the extraction process. Among the examples provided, the most utilized solvents have been methanol (MeOH), acetone (AcO) and ethanol (EtOH), which have been applied at different percentages. Other studies have been performed using alternative options such as water (W), hexane (Hx), chloroform (Ch), dichloromethane (DCM), heptane (Hp) or diethyl ether (DE). According to some investigations, the preferred solvent was EtOH [28,29]. However, in a two-level full factorial design for the extraction of fucoxanthin from *S. siliquosum* and *S. polycystum*, the best solvent was MeOH. In this study, the optimized parameters were temperature (4–45 °C), time (30–1440 min), and solvent to solid ratio (10–50 mL/g), with extraction time. The best conditions were achieved at 30 min and a 45 °C of temperature with a low solvent to solid ratio of 5 mL/g. Fucoxanthin content obtained for *S. siliquosum* and *S. polycystum* was 491.47 and 449.90 µg/g dry weight (DW) when using EtOH and 706.98 and 521.34 µg/g DW for MeOH, respectively [30]. Similarly, the effect of incubation time, temperature, pH, and percentage of solvent was analyzed in a study based on nine different brown algae species [31]. The optimization determined that the yield was maxim at 30 °C for 36.5 min, pH5.7, using 62.2% AcO. In this study, the efficiency of conventional extraction methods was limited by the presence of branched, sulfated or complex polysaccharides (alginate, laminarin, etc.) in algal cell walls [31].

#### 2.1.2. Vortex Assisted Extraction (VAE)

Recently, a study conducted with several brown macroalgae used and validated a VAE system, based on the traditional solid/liquid extraction, whose efficiency is improved by stirring. The results showed that the optimal yield was achieved by using 25 mg of the sample with 300 μL of EtOH and vortexed for 15 min [32].

#### 2.1.3. Soxhlet Assisted Extraction (SAE)

This system is a dynamic process based on continuous reflux of solvent, generally used for health, food and environmental analysis. Temperature is a key factor when it comes to extraction, thus its increase is related to a better extraction since it can break interactions among molecules and facilitate the extraction [33]. SAE with heat application offers an alternative for ME with lower solvent consumption as it allows recircularization. A recent study showed an extraction yield of 0.45 mg/g for the macroalgae *Saccharina japonica*, using n-hexane (n-Hx) as solvent at 40 °C for 16 h [34]. Another study with *Undaria pinnatifida* displayed that the best conditions in SAE were 12 h at 78 °C with EtOH resulting in a yield of 50 µg/g [19].

### 2.2. Non-Conventional Techniques

On the other hand, advances in green technologies currently offer a wide spectrum of solid/liquid procedures that are quite useful for the extraction of the compounds of interest. Some of these emerging technologies involve microwave-assisted extraction, supercritical fluid extraction or ultrasound-assisted extraction, among others. All these methods can be referred to as non-conventional extraction techniques.

#### 2.2.1. Enzyme-Assisted Extraction (EAE)

The main advantage of this method is the ability of enzymes to break the cellulose (hydrolysis) walls of the algae, favoring the accessibility to pigments. Moreover, it is a green technology with no toxic waste and is relatively cheap regarding the cost-effectiveness of the enzymes. A study with *Fucus vesiculosus* shows that by using the enzyme Viscozyme, the best conditions were enzyme-to-W ratio 0.52%, seaweed-to-W ratio 5.37% and enzyme incubation time 3 h. These conditions allowed 0.657 mg/g DW of fucoxanthin to be obtained [35]. Almost a complete recovery of fucoxanthin (96%) extraction was achieved through *U. pinnatifida* by using an enzymatic pre-treatment and then dimethyl ether (DME)+EtOH. The optimum parameters for the pre-treatment were 37 °C at pH6.2 for 2 h, 5% (*w*/*v*) solids, with 0.05% weight enzyme using continuous mixing [36].

#### 2.2.2. Microwave-Assisted Extraction (MAE)

This technique has proved to be suitable for the extraction of bioactive compounds from algae as a viable alternative. A study employed MAE to extract fucoxanthin from *L. japonica*, *U. pinnatifida*, and *S. fusiforme*, using different solvents. Among them, EtOH and Ac obtained the best yields. For safety reasons, EtOH was chosen for further analysis. The extraction conditions were solvent to sample ratio of 10:1 (mL/g) at 50 °C for 10 min. The yields obtained were 5.13, 109.30, and 2.12 mg/100 g, for each algae, respectively [37]. The patent with reference CN104327017A claims a microwave-assisted extraction using EtOH as a solvent in only 5 min, being able to easily perform large-scale production. Then, a liquid-liquid extraction and column chromatography two-step separation process are applied to effectively separate fucoxanthin from chlorophyll and other impurity components.

#### 2.2.3. Ultrasound-Assisted Extraction (UEA)

This technique is considered to be cost-effective and efficient, which utilizes ultrasounds to create micro-bubbles inside the solvent. The growth and collapse of the bubbles cause the breakdown of the macroalgae cell wall, favoring the penetration of the solvent. This technique has been employed to extract fucoxanthin from *Padina tetrastromatica*. The optimized values for solvent concentration, temperature and time were EtOH 80%, 50 °C, and 30 min, respectively. These conditions allowed 750 µg/g DW of fucoxanthin to be obtained, which was higher than the fucoxanthin content obtained with conventional extraction [20].

#### 2.2.4. Pressurized Liquid Extraction (PLE)

This technique is based on applying high temperatures and pressures using a liquid solvent. It is also a green method, as it uses low quantities of solvent and short times. A study performed with the brown algae *Eisenia bicyclis* was done with this technique. It was observed that the parameters that influenced it were temperature and EtOH concentration, with 110 °C and 90% EtOH as the optimal values, obtaining a yield of 0.42 mg/g [38].

#### 2.2.5. Supercritical Fluid Extraction (SFE)

This type of non-conventional extraction requires the fluid to reach a temperature and pressure above the critical point. Among its advantages, some of them can be highlighted as it has great extraction selectivity, short processing times, requires minimal solvents and a low degradability of the extracted product. The most used solvent is carbon dioxide (CO_2_) due to its thermodynamics and heat transfer properties. Moreover, it has a low critical point (31 °C, 73 bar). A co-solvent can be used to modify the extractant polarity. The most important parameters are temperature, pressure and co-solvent [39]. SFE has been applied to *U. pinnatifida*, using EtOH as co-solvent and different ranges of temperature (from 30–60 °C) and pressure (from 80–300 bar) were tested. Higher temperatures (50 °C) and pressures (200 bar) provided a value of 7.53 ng/g DW [40]. Another study based on *U. pinnatifida* showed that the two best conditions for SFE were, for the approach without entrainer, 70 °C and 400 bars for 3 h, which allowed a fucoxanthin yield of 60.12 µg/g to be obtained. The most efficient extraction method required the use of EtOH as entrainer for very similar conditions (60 °C and 400 bar for 3 h) and allowed a fucoxanthin yield of 994.53 µg/g to be achieved [19]. The functionality of this technique also allowed extracting rich-fucoxanthin oil fractions from two brown seaweeds, *S. japonica*, and *S. horneri*. The optimized conditions consisted of a flow rate of 27 mg/min of CO_2_ at a temperature of 45 °C and a pressure of 250 bar for 2 h. Under these parameters, the concentration of fucoxanthin was 0.41 ± 0.05 mg/g for *S. japonica* and 0.77 ± 0.07 mg/g for *S. horneri*. The SFE extracts showed greater in vitro activity than the extracts obtained with other techniques [41].

### 2.3. Comparison of Extraction Systems

The main objective of the comparison is to elucidate the most efficient extraction methods in terms of recovery. As observed in Table 1, a great variability among brown macroalgae extracted, different solvents, and different extraction conditions exist. Taking this into account, it is very complex to perform a comparison. When possible, studies using the same species, but different extraction systems were selected and further discussed in this section. *U. pinnatifida* has been the most studied macroalgae species and has been extracted using different techniques: MAE, PLE, SFE, and ME. The highest values reported were 2.671 mg/g DW and 4.96 mg/g fresh weight (FW) using ME [21,22]. *Laminaria japonica* and *S. fusiforme* have been extracted by ME and MAE. As in the previous case, the conventional ME allowed a greater content to be obtained (0.19 and 6.62 mg/g DW for *L. japonica* and *S. fusiforme*, respectively) compared to MAE (0.04 and 0.06 mg/g DW, respectively) [37,42,43]. CE and SFE systems have been utilized with the macroalgae species *F. serratus* and *S. horneri*. For *F. serratus*, the studies reported quite similar fucoxanthin content, 3.57 and 2.18 mg/g DW for ME and SFE, respectively. Regarding *S. horneri*, yield differences were more pronounced, obtaining 4.49 and 0.77 mg/g DW of fucoxanthin using ME and SFE, respectively [27]. *F. vesiculosus* has been extracted by VAE and EAE, both techniques showing similar fucoxanthin recovery values: 0.70 and 0.66 mg/g DW, respectively [31,35]. Several studies have extracted fucoxanthin from *Dictyota dichotoma* using ME and VAE. In this case, the differences in the extraction yield were noticeable, since ME extracted 6.42 mg/g DW of fucoxanthin, versus 0.60 mg/g DW obtained with VAE. Finally, *P. tetrastromatica* has been extracted by using ME and UAE. Both techniques extracted a similar content of fucoxanthin, specifically, 0.41 and 0.75 mg/g DW [20,44].

Concluding, the ME has been used in a great variety of brown algae and, in general, is the best technique in terms of fucoxanthin recovery. Conventional techniques are low cost and present lower performance difficulty [45]. However, in several cases, the extraction times are long (which may cause deterioration of fucoxanthin) and the solvents used are toxics, such as MeOH. Compared to ME, few studies have been performed using the non-conventional techniques mentioned in this study. According to the scientific literature performed, SFE might be the best non-conventional technique, in terms of extraction efficiency. One of the advantages of this system is the use of CO_2_ as a solvent, an easy-available compound and non-toxic. Although more studies are still needed, SFE systems could be an efficient and respectful option for the industrial production of fucoxanthin.

## 3. Quantification, Identification, and Purification Methods

### 3.1. Quantification and Identification of Fucoxanthin

Fucoxanthin is a pigment that offers different alternatives for its quantification since it can be performed through analytical techniques (based on its molecular mass detection), spectrophotometric methods (based on its color feature), or a combination of both systems. The most common instruments utilized for its identification and quantification are: (1) liquid chromatography-mass spectrometry (LC-MS) using mass-to-charge ratio (*m*/*z*); (2) high-performance liquid chromatography (HPLC) coupled to ultraviolet (UV) or (photo)diode array detectors (PDA/DAD), which detects maximum absorbance peak at 446 nm; (3) nuclear magnetic resonance (NMR), which determines its structure; or (4) spectrophotometric readers that provide absorbance based data [28]. Most of the protocols developed for the quantification of fucoxanthin using LC, either HPLC or UPLC equipment, usually establish absorbance around 450 nm. For instance, samples from *Turbinaria turbinata* and *S. plagyophyllum* were analyzed selecting a detection wavelength of 450 nm [56]. However, the presence of conjugated double bonds in the fucoxanthin formula (Figure 1) make it unstable under some conditions. Fucoxanthin can get isomerized into cis-fucoxanthin, whose oxidation may produce short-chain carbonyl compounds. To detect and provide an accurate fucoxanthin quantification, it is useful to perform wide spectra readings (from 300 to 500 nm) or establish the specific wavelengths associated with these sub-products (300, 350 and 400 nm) [60]. Regarding LC-MS, the quantification of the fucoxanthin has been mostly performed by LC-MS, using either electrospray or atmospheric pressure chemical ionization (ESI or APCI, respectively) sources and coupled to additional detectors such as UV or DAD, among others [37,52,60,61,62]. Other approaches include different mass detector improvements, like UPLC-PDA-TWIMS-QTOF-MS (UPLC-PDA coupled to a quadrupole/traveling-wave ion mobility/time-of-flight MS) [63]. Among the spectrometric techniques used for identifying fucoxanthin, Fourier transform infrared (FTIR) has been used in the region of 500 to 4000 cm^−1^ for characterizing purified fractions obtained from extracts of *Himanthalia elongata*. In the same study, an UV-visible detector coupled to DAD (190 to 600 nm) also provided spectroscopy results [64]. NMR techniques are very useful tools for the identification and the structural determination of fucoxanthin and its sub-products. In fact, it has been used as a confirmatory instrument in many fucoxanthin-related works [4,21,37,52,61,65,66,67,68]. Finally, spectrophotometry techniques have been widely utilized for the identification and quantification of several pigments, mostly as a sum of carotenoids. To obtain robust data, spectrophotometric methods require the previous adjustment of the quantification protocol to the specific experimental conditions: establish the correct titration of the sample, adapt reading parameters and equations to different solvents, and determine adequate negative and positive controls [44,69,70]. The specific quantification of fucoxanthin by spectrophotometry has been scarcely developed [44,71]. Nevertheless, they have been described as easier, faster and cheaper than LC. A study analyzed the content of fucoxanthin extracts using a microplate reader. This technique allowed results with lower standard errors (<5%) than HPLC to be obtained [71]. Spectrophotometric methods are cheaper since they reduce experimental times, do not require highly trained personnel or equipment for their development, nor do they use a high volume of organic solvents, so they can be also considered as greener techniques than those that are LC-based. The preferred quantification method for fucoxanthin still remains to be those based on the use of HPLC or LC instruments; however cheaper, quicker, and easier options like spectrophotometry have been suggested as a promising screening tool.

### 3.2. Purification

Fucoxanthin-rich extracts may be concentrated and further purified. For the performance of this step, extracts can be loaded onto a silica gel packed into a glass column and with a solvent. The solvent type varies throughout scientific literature; however, n-Hx is frequently used as part of a mixture of solvents, for example, n-Hx:AcO, which has been applied in different proportions such as 6:4 (*v*:*v*) and 7:3 (*v*:*v*) [28,72]. Another study used a few solvents starting with a mixture of n-Hx:DE that gradually reduced the amount of n-Hx from 80 up to 0, then finished with MeOH. This option showed a co-elution of lipids. Thus, to achieve a higher degree of purity, fractions were dissolved in Ch and applied on preparative thin-layer chromatography plates using a combination of Hx/De/acetic acid (70:30:1, volume-based) and MeOH. To separate neutral lipids, the latter solution may be used, and for the polar ones, Ch/AcO/MeOH/acetic acid/W (50:20:10:10:5, volume-based) [63]. Similarly, another work used Ch/DE/n-Hx/acetic acid (10:3:1:1, volume-based) to obtain a purified extract of fucoxanthin [64]. Another method of purification is based on salt aqueous two-phase systems, but this process needs additional purification steps. An effective alternative is the use of ultra-filtration to process an ethanolic salt aqueous two-phase system which may reduce the unspecific losses of the pigment as well as the amount of protein impurities, allowing an increase in the purity of the permeate up to 63% [73]. After the selected purification step, the orange-red fraction that contains fucoxanthin is collected and can be submitted to further purification stages with a process like prep-HPLC. After its separation, the fraction of interest is further analyzed to identify it.

## 4. Molecule Stability

Once fucoxanthin has been extracted, it is fundamental to store it under stable conditions, paying special attention to its exposure to light, extreme pH value or temperature. This section offers an overview of the stability studies performed with fucoxanthin. Three different approaches, which are presented below in increasing order of complexity, have been developed to confirm the chemical stability of the molecule. The evaluation of fucoxanthin has been performed of the free compound, embedded as part of an emulsion, or encapsulated.

### 4.1. Free Molecule

The unique chemical characteristics of fucoxanthin (unusual allenic bond, epoxide group, and conjugated carbonyl group within a polyene chain) (Figure 1) confer several oxidation targets to this molecule. The main factors that can trigger carotenoids degradation are oxygen, light, high temperatures, enzymatic reactions, heavy metals exposure, and extended periods of storage [74,75,76,77,78,79]. Different studies have evaluated the stability of fucoxanthin in terms of temperature when freely added to diverse matrixes. The stability of free fucoxanthin in water and milk was observed to decrease with the increment of the temperature. At 2 °C, fucoxanthin was stable for 28 days in milk. After 28 days at 10 and 26 °C, fucoxanthin stability in milk was reduced by about 20%. In water, the compound showed a degradation of nearly 40 and 80%, at 10 and 26 °C, respectively [80]. However, other experiments showed no significant differences in rich-fucoxanthin extracts stored at 4 °C or 25 °C (*p* > 0.05) for 4 weeks. The treatment at 50 °C temperature displayed a significant decrease in the stability of the fucoxanthin after the third week of storage [65]. Similar results were obtained when fucoxanthin was subdued to 75 °C for 60 min, showing a stability loss of 94%. At 25 °C, this level of stability loss is reached between the fourth (87%) and the ninth (99%) day [74]. Other works have evaluated fucoxanthin stability in terms of pH. A stability study analyzed extracts obtained from *S. binderi* treated at different pH conditions. The initial pH (6.1) of the fucoxanthin-rich extract was shifted to both very acidic (pH of 1 and 3) and very alkaline (pH of 9, 11 and 13) environments. It was found that the ideal range to extend fucoxanthin stability is in a pH between 5 and 7 [65]. The chemical behavior of free fucoxanthin was also evaluated under a digestibility in vitro model where the samples were exposed to different molecules and pH changes (from 2.2 to 7.0). The results showed a progressive degradation from the simulated stomach conditions (10%) to the ileum (20%) accompanied by a transformation into fucoxanthinol. This metabolite represented more than 50% of the fucoxanthin in the duodenum and nearly 100% in the ileum [75]. Finally, light has been also considered in the stability determination. Fucoxanthin is a pigment that has been demonstrated to be sensitive to light exposure. When free fucoxanthin is preserved at darkness the molecule is more stable than when exposed to light. Under light the stability drops more than 50% after 4 days of storage [65]. Other work that utilized longer storage periods confirmed that keeping fucoxanthin in darkness extended its shelf-life with a degradation rate lower than 10% [81]. Therefore, the optimal storage conditions for free fucoxanthin include its conservation in darkness, in a solution with a pH between 5 and 7, and at low temperatures, preferably those lower than room temperature.

### 4.2. In Emulsions

The inclusion of ingredients in emulsions is usually considered a good option to achieve the stabilization of the compound. Food grade emulsions consist of creating small lipid droplets dispersed in water that may be utilized for incorporating different molecules of interest into fatty food matrixes. The inclusion of the biomolecules into the droplets improves their bioavailability since it preserves their bioactivities and their chemical features. A study studied the fucoxanthin stability when incorporated into oil-in-water emulsions using different natural emulsifiers (whey protein isolate, lecithin and Arabic gum). Among the tested emulsifiers, whey protein was the one that provided better stability to fucoxanthin. This emulsifier slowed down the degradation of fucoxanthin, displaying a 30% loss after 15 days of storage at 25 °C while Arabic gum and lecithin emulsions showed a 60% degradation in the first five days [82]. In another study, fucoxanthin was loaded into emulsions using carrier oils with different properties: oils with long and medium-chain triacylglycerols and indigestible oils. This study concluded that fucoxanthin was more soluble and got absorbed better when included into micelles created with oils containing long and medium-chain triacylglycerols. However, any of the tested emulsions provide similar fucoxanthin concentrations (being fucoxanthinol and amarouciaxanthin A quantified as fucoxanthin equivalents) when estimated in serum samples [83]. Finally, other research performed a further analysis of fucoxanthin after its emulsification in an oil/water system. It showed that total fucoxanthin and all its trans-, 13-cis and 13’-cis isomers suffered a significant degradation when stored at different temperatures. The stability decrease was stronger when fucoxanthin-like molecules were subdued to 60 °C, since the nearly total loss was detected after the fifth day of storage. This degradation rate was slower when applying temperatures of 5 °C and 37 °C showing 80% losses after 40 of 14 storage days, respectively. Total and all-trans fucoxanthin suffered degradation with temperature dependence and promoted the formation of complexes of activated structures, which are more sensitive to degradation. When samples were exposed to light and pH was reduced, the degradation rates were much more dramatic but promoted the formation of the 9’-cis isomer. This compound was the only one that was able to resist these treatments in the emulsion, except at low values of pH. It increased its concentration under neutral pH of 7.5 or light exposition since the isomerization reaction predominated over the degradation one induced by oxidation, protonation or illumination. In actuality, the degradation rate of the 9’-cis isomer was lower than for fucoxanthin and its isomers (all-trans, 13-cis, and 13’-cis) in the emulsion. In this study, the stability of fucoxanthin-like molecules in emulsions was deeply analyzed, showing that the extrinsic factors determining their degradation were established from greater to lower influence: pH, temperature and exposure to light [84]. Therefore, further studies should be conducted to disclose the effect of different emulsifiers or carrier oils on the stability of fucoxanthin isomers. Nevertheless, their addition seems to be required to prolong the stability of fucoxanthin-like molecules when incorporated into emulsions.

### 4.3. Encapsulation

Encapsulation is a process that provides a physical barrier to prevent the alteration of the core ingredient. This technique stabilizes and protects molecules that get easily degraded, hence encapsulation allows the fortification of different matrixes with compounds that otherwise would have lost their bioactivities. The stability and bioavailability of fucoxanthin when encapsulated using different approaches and materials has been evaluated. The variability of wall materials that have been used for developing capsules is huge. Different proteins (whey, zein, casein and gelatin), oligosaccharides (maltodextrins, cyclodextrin), polysaccharides (chitosan, alginates) or glycolipids are commonly employed as encapsulation materials, applied both individually or combined. A study evaluated up to six ingredients to microencapsulate fucoxanthin: hydroxypropyl-β-cyclodextrin, maltodextrin, gum Arabic, whey protein isolate, isolated pea protein and gelatin. After exposing microcapsules to a temperature of 90 °C for 24 h, those built with whey protein isolate, gum Arabic, and maltodextrin displayed the lower degradation rates with values of 38%, 44% and 45%, respectively [76]. Another work utilized nanogels prepared by ionic gelation with different amounts of chitosan and glycolipid or sodium tripolyphosphate. The analysis performed with an FTIR revealed that fucoxanthin creates numerous hydrogen bonds with chitosan. Results obtained from X-ray tests showed that fucoxanthin gets arranged in a disorderly manner within the chitosan-nanogels. However, fucoxanthin remained more stable when introduced into nanogels formed by the combination of chitosan with glycolipid. The presence of glycolipid improves its stability, prolongs the storage time and avoids its degradation up to 45 h. Regarding the biological availability of fucoxanthin, an in vitro test demonstrated that the use of chitosan-glycolipid nanogels offered the highest bioavailability rate (68%), followed by chitosan (51%) and glycolipid nanogels (35.5%) [85]. Another work compared the efficiency of fucoxanthin encapsulated into casein nanoparticles and the same nanoparticles but with a chitosan coat. Both kinds of particles were submitted to a simulated digestion process in an in vitro assay where they were presented to different enzymes and fluid secretions (α-amylase, pepsin, bile extract, pancreatic lipase, and pancreatin) and pH changes (from 6.5 to 2.2, then to 5.5, slowly increased to 6.0 and 7.0). The pass of fucoxanthin through the gastrointestinal tract has been demonstrated to trigger its transformation into fucoxanthinol. Fucoxanthin contained into the nanoparticles also get transformed into fucoxanthinol. However, those additionally coated with chitosan slowed down the process and also provided slight degradation rates (20%) that were mostly observed during the steps simulating the jejunum and ileum [75]. Another approach also based on the use of casein (sodium caseinate) showed the advantages of mixing it with zein to create nanoparticles (100 to 130 nm). Both components of the nanocapsules interacted with fucoxanthin by hydrophobic contacts, which seemed to stabilize the molecule and its bioactivities since after heating fucoxanthin (75 °C) it remained stable for 60 min, showing an estimated 20% degradation after 140 min. Other nanoparticles created with just caseinate or just zein showed about 15% and 30% degradation rate after 60 min, respectively. The caseinate-zein nanocapsules also extend the stability of fucoxanthin when stored at 25 °C for further than 16 days showing at that time a degradation rate lower than 30% [74]. Therefore, encapsulation allows the isolation of the target ingredients, providing chemical stability, which ultimately will preserve their bioactivities, and also allows the determination of an exact dosage. The incorporation of exact concentrations into a matrix may represent a comfortable administration format to the consumers, which will allow the maximization of health benefits associated with fucoxanthin which are mostly dose-dependent. Hence, the encapsulation of fucoxanthin is an accurate method to specify its final amount, facilitating the potential adherence for fucoxanthin-based treatments [86].

## 5. Developed Products Containing Fucoxanthin and Their Health Benefits

**Figure 2:**
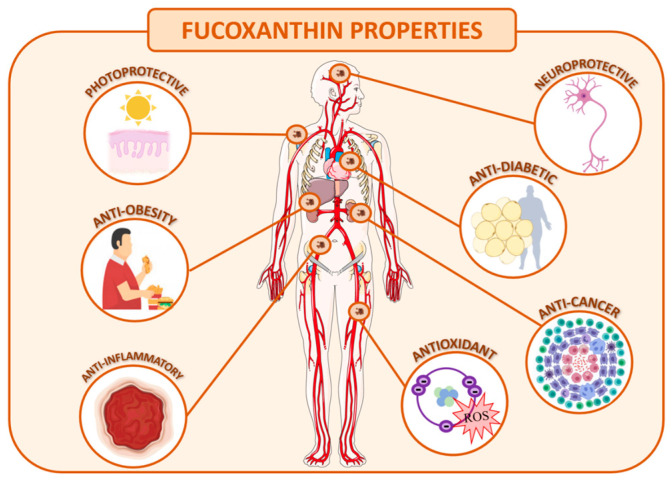
Human health benefits of fucoxanthin.

The optimization of the extractive process, purification and stabilization of this molecule is of great interest to the industry due to the several therapeutic activities described, making it a useful compound to incorporate in nutraceuticals, cosmetics, and even pharmaceutical products. As mentioned before, fucoxanthin has gathered much attention of late due to its strong antioxidant proprieties, anti-cancer, anti-inflammatory activities and anti-obesity effects, among others (Figure 2) [87]. Regarding the last property, fucoxanthin can be added as a food component to accelerate the adaptive thermogenesis [88]. *U. pinnatifida* lipids, containing 9.6% fucoxanthin, significantly reduced the weight of abdominal white adipose tissue of rats and mice. Bodyweight of mice fed with 2% of *Undaria* lipids was significantly lower than that of controls [89]. These results suggest that fucoxanthin could be used in dietary supplements with anti-obesity potential, to treat or prevent diseases related to excessive weight. Currently, these supplements can be found in the market, in the form of oil or as a microencapsulated powder with the commercial name of ThinOgen^®^ and Fucovital^®^, for example. They are supposed to help weight loss and also improve eye, brain, liver, and joint health [90]. Furthermore, fucoxanthin may be used to combat neurodegeneration. The neuroprotective proprieties of this molecule were tested in models of traumatic brain injury. In vitro studies demonstrated that fucoxanthin increased neuron survival and reduced the reactive oxygen species levels [91]. It was observed that the administration of fucoxanthin in cerebral ischemic/reperfusion injury models suggested that this pigment could be exploited as a therapeutic target for protecting neurons [92]. These results point out that the use of fucoxanthin as a nutritional supplement could be interesting for the prevention or even the treatment of brain injuries and neurodegenerative pathologies. Some studies conducted about this are already incorporating fucoxanthin in several foods, such as enriched canola oil [93], fortified yogurt [94], milk [95], baked products like scones [96] and even ground chicken breast meat [97].

In the cosmetics and pharmaceutical industries, this carotenoid is also relevant, due to its skin protective effects against burns and filaggrin (filament aggregating protein) disorder induced by radiation. Fucoxanthin demonstrated great results against UV radiation and was able to protect the dermal layers. This dermoprotective capacity can be explained through the promotion of filaggrin which generates a dermal barrier [98]. This protection has also been associated with the remediation of DNA damage and the potent antioxidant activity of fucoxanthin in human fibroblast cells [99]. An inhibitory effect on tyrosinase activity in guinea pigs exposed to UV radiation was also reported, showing a decrease in the harmful effects. Other study demonstrated that oral administration of fucoxanthin produced a suppression of the transcription of the melanogenesis factor, due to the inhibition of dermal mRNA expression related to this disease [100]. These results seem to indicate that the oral or topical administration of fucoxanthin can prevent or even reduce the negative effects induced by UV radiation exposure, such as the appearance of melanomas. In fact, due to its antioxidant capacity, fucoxanthin has been tested in several anti-aging and anti-sun cosmetic formulations [101,102].

## 6. Future Perspectives and Conclusions

Fucoxanthin is considered a valuable molecule due to its wide range of beneficial properties, like antioxidant activity, cardiovascular protection, neuroprotective effect or photoprotective properties, among others. All these turn fucoxanthin into an interesting pigment with promising industrial applications in the food, cosmetic and pharmaceutical sectors. Nevertheless, the commercialization of fucoxanthin is scarce, which greatly limits its further use. As mentioned before, fucoxanthin may be chemically synthesized, but this process is inefficient and complex, while the extraction method from marine organisms has not been standardized. Therefore, it is necessary to design a practical way to profit from its properties. First would be the extraction of fucoxanthin from brown macroalgae, as there is easy and economic access to these organisms, and it avoids the safety issues prompted using chemical compounds. However, the extraction yield of fucoxanthin has been found to be very variable depending on the selected species and the recovery technique, as demonstrated by the data provided from the analyzed scientific literature. In this context, further studies are needed to standardize the fucoxanthin obtaining process. The application of methods like response surface methodology is likely to optimize extraction conditions for fucoxanthin. Moreover, even though conventional techniques are widely employed, non-conventional extraction techniques are gaining importance and new approaches are expected. Once fucoxanthin has been extracted, it is fundamental to store it under stable conditions, paying special attention to its exposure to light, extreme pH values, or temperatures. The encapsulation of fucoxanthin has been revealed to be the most profitable technique for the conservation of fucoxanthin-like molecules. Nowadays, different products have been fortified with fucoxanthin and proved to exert different health benefits (such as loss weight loss or antioxidant capacity, among others).

In conclusion, the selection of the extraction and purification methods is a key factor to achieve acceptable yields, prevent pigment degradation and reduce production costs. In addition, the use of environmentally friendly extraction/purification techniques may increase the commercial value of the final product. Among the different extraction methods provided in this article, it is necessary to standardize the experimental conditions, especially when scaling up the process to an industrial level. Regarding the storage alternatives, fucoxanthin encapsulation using natural products is considered as one of the best approaches, followed by its emulsion using natural emulsifiers. Therefore, future and innovative studies regarding efficient, quick, eco-friendly and safe extraction methods can speed up the progress towards its commercialization and incorporation of fucoxanthin in the global market.

## Figures and Tables

**Table 1:** Brown algae species described as a source of fucoxanthin extracted by conventional extraction techniques, detection methods employed for determining its presence and quantification values (fucoxanthin concentration are expressed in fucoxanthin (Fx) mg/g of DW, or where not determined, “nd” was indicated).

Algae Species	Solvent	Extraction Conditions	Detection Method	Fx (mg/g DW)	Ref.
**ME**					
*Alaria crassifolia*	MeOH	RT, 12 h	HPLC-PDA	1.10	[46]
*Alaria esculenta*	AcO 62.2%	30 °C, 36.5 min	HPLC-DAD	0.87	[31]
*Analipus japonicas*	MeOH	RT, 12 h	HPLC-PDA	1.40	[46]
*Cladosiphon okamuranus*	MeOH	RT, 1 h	HPLC-DAD	0.27	[47]
*Cystoseira hakodatensis*	Ch, MeOH (1:2)	RT, 2 h	HPLC-DAD	2.01	[48]
	MeOH	RT, 12 h	HPLC-PDA	2.40	[46]
	Ch/MeOH (1:2)	RT, 1 h	HPLC-DAD	3.47	[24]
*Desmarestia viridis*	MeOH	RT, 12 h	HPLC-PDA	0.10	[46]
*Dictyopteris australis*	AcO	4 °C, 12 h	Spec	0.23	[44]
*Dictyota dichotoma*	EtOH	RT, 15 min × 5	HPTLC	0.44	[49]
	AcO	4 °C, 12 h	Spec	0.18	[44]
	MeOH	RT, 24 h	HPLC-PDA	6.42	[50]
*Ecklonia kurome*	Chl/MeOH (1:2)	RT, 2 h	HPLC-DAD	1.68	[48]
*Fucus distichus*	MeOH	RT, 12 h	HPLC-PDA	0.90	[46]
	AcO	RT, 5 min	Spec	0.16	[51]
*Fucus serratus*	Hx/AcO (70:30)	RT, 24 h	HPLC-DAD	3.57	[27]
*Himanthalia elongata*	n-Hx, DE, Ch	RT, 15 min	LC-ESI-MS, HPLC, ^1^H-NMR	18.60	[52]
*Hizikia fusiformis*	MeOH	-	HPLC-DAD	0.02	[43]
*Ishige okamurae*	MeOH		HPLC-DAD	nd	[53]
*Iyengaria stellate*	AcO	4 °C, 12 h	Spec	0.18	[44]
*Kjellmaniella crassifolia*	MeOH	RT, 15 min	HPLC-DAD	0.15	[54]
*Laminaria japonica*	MeOH	-	HPLC-DAD	0.19	[43]
*Laminaria digitata*	AcO 62.2%	30°, 36.5 min	HPLC-DAD	0.65	[31]
*Laminaria religiosa*	MeOH	RT, 96 h	HPLC-DAD, ^1^H-NMR, ^13^C-NMR	0.24	[21]
*Laminaria saccharina*	AcO	RT, 5 min	Spec	0.24	[51]
*Leathesia difformis*	MeOH	RT, 12 h	HPLC-PDA	0.30	[46]
*Lobophora variegata*	AcO	4 °C, 12 h	Spec	0.23	[44]
*Melanosiphon intestinalis*	MeOH	RT, 12 h	HPLC-PDA	1.90	[46]
*Myagropsis myagroides*	MeOH	RT, 24 h	HPLC-PDA	9.01	[50]
*Padina australis*	Chl/MeOH (1:2)	RT, 2 h	HPLC-DAD	1.29	[48]
*Padina gymnospora*	AcO	4 °C, 12 h	Spec	0.43	[44]
*Padina minor*	EtOH	RT, 15 min × 5	HPTLC	0.50	[49]
*Padina pavonica*				0.43	
*Padina tetrastromatica*	AcO	4 °C, 12 h	Spec	0.41	[44]
*Petalonia binghamiae*	MeOH	RT, 48 h	HPLC-DAD, ^1^H-NMR, ^13^C-NMR	0.58	[21]
*Saccharina japonica*	MeOH	RT, 15 min	HPLC-DAD	0.03	[54]
*Saccharina sculpera*	MeOH	RT, 12 h	HPLC-PDA	0.70	[46]
*Sargassum binderi*	MeOH	RT, 12 h × 2	HPLC-DAD	0.73	[55]
*Sargassum confusum*	MeOH	RT, 12 h	HPLC-PDA	1.60	[46]
*Sargassum crassifolium*	Chl/MeOH (1:2)	RT, 2 h	HPLC-DAD	1.64	[48]
*Sargassum duplicatum*	MeOH	RT, 12 h × 2	HPLC-DAD	1.01	[55]
*Sargassum fulvellum*	MeOH	-	HPLC-DAD	0.01 *	[43]
*Sargassum fusiforme*	MeOH	RT, 12 h	HPLC-PDA	1.10	[46]
	AcO/EtOH (1:40)	65 °C, 80 min	Spec	2.62	[42]
*Sargassum horneri*	Chl/MeOH (1:2)	RT, 2 h	HPLC-DAD	2.12	[48]
	MeOH	RT, 12 h	HPLC-PDA	3.70	[46]
	Ch/MeOH (1:2)	RT, 1 h	HPLC-DAD	4.49	[24]
*Sargassum linearifolium*	AcO	4 °C, 12 h	Spec	0.37	[44]
*Sargassum muticum*	AcO	RT, 5 min	Spec	0.29	[51]
*Sargassum plagiophyllum*	AcO/MeOH (7:3)	ice, 15 min	HPLC	0.71	[56]
*Sargassum polycystum*	EtOH	RT, 15 min × 5	HPTLC	0.41	[49]
*Sargassum siliquastrum*	Chl/MeOH (1:2)	RT, 2 h	HPLC-DAD	1.99	[48]
*Sargassum thunbergii*	MeOH	RT, 12 h	HPLC-PDA	1.80	[46]
*Scytosiphon lomentaria*	MeOH	RT, 12 h	HPLC-PDA	0.50	[46]
	MeOH	RT, 96 h	HPLC-DAD, ^1^H-NMR, ^13^C-NMR	0.56	[21]
*Silvetia babingtonii*	MeOH	RT, 12 h	HPLC-PDA	0.70	[46]
*Spatoglossum asperum*	AcO	4 °C, 12 h	Spec	0.58	[44]
*Sphaerotrichia divaricata*	MeOH	RT, 12 h	HPLC-PDA	0.20	[46]
*Stoechospermum marginatum*	AcO	4 °C, 12 h	Spec	0.37	[44]
*Turbinaria ornate*	Chl/MeOH (1:2)	RT, 2 h	HPLC-DAD	1.27	[48]
*Turbinaria spp.*	AcO	4 °C, 12 h	Spec	0.43	[44]
*Turbinaria turbinata*	AcO/MeOH (7:3)	ice, 15 min	HPLC	0.59	[56]
*Undaria pinnatifida*	MeOH	RT, 96 h	HPLC-DAD, ^1^H-NMR, ^13^C-NMR	2.67	[21]
	MeOH	RT, 1 h	HPLC-DAD	2.08	[22]
	MeOH	RT, 1 h	HPLC-DAD	4.96 *	
	EtOH	RT, 1 h	HPLC-DAD	0.70	[36]
**VAE**					
*Ascophylum nodossum*	EtOH	RT, 15 min	HPLC-PDA	0.02	[32]
*Dictyota dichotoma*				0.60	
*Fucus vesiculosus*	EtOH	RT, 15 min	HPLC-PDA	0.02	[32]
	AcO	40 °C, 40 min	HPLC-DAD	0.70	[31]
*Sargassum vulgare*	EtOH	RT, 15 min	HPLC-PDA	0.40	[32]
*Zonaria tournefortii*				0.80	
**SAE**					
*Feldmannia mitchelliae*	EA	80 °C, 16 h	HPLC	5.50	[57]
*Saccharina japonica*	n-Hx	40 °C, 16 h	HPLC	0.45	[34]
*Sargassum swartzii C. Agardh*	EA	80 °C, 6 h	FT-IR, ^1^H-NMR, ^13^C-NMR	0.17	[58]
**EAE**					
*Fucus vesiculosus*	W	Viscozyme, 50 °C, 100 rpm, 10 min	HPLC-UV, LC-MS	0.66	[35]
**MAE**					
*Laminaria japonica*	Hp, AcO, W	50 °C, 10 min	LC-ESI-MS, HPLC, ^1^H-NMR	0.04	[37]
*Sargassum fusiforme*				0.02	
*Undaria pinnatifida*				0.90	
**UEA**					
*Padina tetrastromatica*	EtOH	50 Hz, 30 min	HPLC-DAD	0.75	[20]
**PLE**					
*Eisenia bicyclis*	EtOH	110 °C, 5 min	HPLC-PDA	0.42	[38]
*Undaria pinnatifida*	EtOH	78 °C, 12 h	HPLC-UV	0.05	[19]
**SFE**					
*Fucus serratus*	EtOH	50 °C, 304 bars, 1 h	HPLC-DAD	2.18	[27]
*Sargassum horneri*	CO_2_, EtOH	45 °C, 250 bars	HPLC-DAD	0.77	[41]
*Sargassum japonica*				0.41	
*Sargassum muticum*	CO_2_, EtOH	50 °C, 100 bars	HPLC-DAD	0,55	[59]
*Undaria pinnatifida*	CO_2_, EtOH	50 °C, 200 bars	HPLC-UV	<0.01	[40]
	CO_2_, EtOH	60 °C, 400 bars	HPLC-UV	0.99	[19]

Solvent: Ethanol (EtOH); Methanol (MeOH); Acetone (AcO); Chloroform (Ch); Hexane (Hx); n-Hexane (n-Hx); Diethyl ether (DE), Ethyl Acetate (EA); Water (W); Heptane (Hp); Carbon dioxide (CO_2_). Extraction methods and conditions: Maceration extraction (ME); Vortex-assisted solid-liquid micro-extraction (VAE); Room temperature (RT); Soxhlet-assisted Extraction (SAE); Enzyme assisted extraction (EAE); Microwave-assisted extraction (MAE); Ultrasound-assisted extraction (UAE); Pressurized liquid extraction (PLE); Supercritical fluid extraction (SFE). Detection method: High Performance Liquid Chromatography (HPLC); HPLC with diode array detector (HPLC-DAD); HPLC with ultraviolet detector (HPLC-UV); High Performance Thin Layer Chromatography (HPTLC); Liquid Chromatography-Atmospheric Pressure Chemical Ionization coupled to Mass Spectrometry (LC-APCI-MS); Liquid Chromatography-Electrospray Ionization coupled to Mass Spectrometry (LC-ESI-MS); Carbon-13 nuclear magnetic resonance (^13^C-NMR); Proton nuclear magnetic resonance (^1^H-NMR); Spectrophotometry (Spec); Fourier Transform-Infrared Spectroscopy (FT-IR). Fucoxanthin content: not determined: fucoxanthin was found but not quantified (*nd*); Dry Weight (DW); Fresh Weight (* FW).

## Abbreviations

**Generic**	
DW	Dry weight
FW	Fresh weight
RT	Room temperature
**Extraction techniques**	
EAE	Enzyme-assisted extraction
MAE	Microwave-assisted extraction
ME	Maceration extraction
PLE	Pressurized liquid extraction
SFE	Supercritical fluid extraction
VAE	Vortex assisted extraction
**Compounds**	
AcO	Acetone
Ch	Chloroform
CO_2_	Carbon dioxide
DCM	Dichloromethane
DE	Diethyl ether
DME	Dimethyl ether
EtOH	Ethanol
H_2_O_2_	Hydrogen peroxide
Hp	Heptane
Hx	Hexane
MeOH	Methanol
n-Hx	n-Hexane
W	Water
**Detection and purification techniques**	
^13^C-NMR	Carbon-13 nuclear magnetic resonance
^1^H-NMR	Proton nuclear magnetic resonance
APCI	Atmospheric Pressure Chemical Ionization
DAD	Diode-array detector
PDA	Photodiode-array detector
ESI	Electrospray ionization
FTIR	Fourier-transform infrared spectroscopy
HPLC	High-performance liquid chromatography
HPTLC	High Performance Thin Layer Chromatography
LC	Liquid chromatography
MS	Mass spectrometry
NMR	Nuclear Magnetic Resonance
QTOF	Quadrupole time of flight
Spec	Spectrophotometry
TWIMS	Traveling-wave ion mobility MS
UPLC	Ultrahigh-performance liquid chromatography
UV	Ultraviolet
